# 3D imaging of left atrial dissection: a case report

**DOI:** 10.1186/s44215-023-00095-6

**Published:** 2023-08-01

**Authors:** Ryoi Okano, Dai Kawashima, Yusuke Koshiba, Kimiaki Ai, Takuya Ando, Satoshi Suzuki, Satoru Maeba

**Affiliations:** 1 Department of Cardiovascular Surgery, Takada General Hospital, Aizuwakamatsu, Japan; 2 Department of Anesthesiology, Takeda General Hospital, Aizuwakamatsu, Japan; 3 Department of Cardiology, Takeda General Hospital, Aizuwakamatsu, Japan; 4 Department of Cardiovascular Surgery, Tokyo General Hospital, Tokyo, Japan

**Keywords:** Left atrial dissection, Computed tomography, Transesophageal echocardiography, Complication

## Abstract

**Background:**

Left atrial dissection (LAD) is an uncommon but potentially devastating complication of cardiac surgery. Though surgical and conservative treatment strategies have been reported, the choice depends on each clinical situation. Especially in sensitive cases, the decision could be difficult, where the detailed assessment of the multiple imaging modalities is mandatory.

**Case presentation:**

Open surgical total arch replacement (TAR) was performed on a male patient aged 79 years old, who had severe chronic obstructive pulmonary disease (COPD) and a history of aortofemoral bypass for abdominal aortic aneurysm and arteriosclerosis obliterans (ASO). During the weaning off the cardiopulmonary bypass (CPB), LAD was detected on intraoperative transesophageal echocardiography (TEE). It was 18 × 26 mm and full of hematoma with the TEE. Due to the patient’s frailty and not to elongate the CPB duration, we selected a conservative strategy. The patient was extubated on postoperative day (POD) 1 and transferred from ICU to the ward on POD 3. On POD 7, ECG-gated 3D-CT was performed, on which LAD occupied 26% of the left atrial volume. It also revealed the opening of the pulmonary veins and the proximity of the LAD and the coronary sinus (CS). The cause of the LAD was considered to be the CS perforation with a retrograde cardioplegic cannula. A follow-up 3D-TEE was performed on POD 15, where the hematoma inside the LAD was absorbed. He was discharged home at POD 23. With transthoracic echocardiography, LAD itself disappeared after 3 months.

**Conclusion:**

3D imaging, such as 3D-TEE and 3D-CT, is valuable in the assessment of the volume and quality of LAD. Furthermore, it clarifies the exact position and configuration of LAD, which help in assessing the etiology, predicting the hemodynamic disturbance, and determining the treatment strategy. They are potent tools, especially in complex cases.

## Background

Left atrial dissection (LAD) is a detachment of the intima from the left atrial wall, which makes a sizeable false lumen space and a massive contained hematoma in it. It may cause devastating hemodynamic disturbance and subsequent poor results [1–3]. LAD mainly occurs in mitral valve surgery and could develop in other cardiac procedures, such as coronary artery bypass grafting (CABG), left ventricular aneurysmectomy, pulmonary vein cannulation, and cardiac mass excision [1, 3]. Damage to the atrioventricular (AV) groove and coronary sinus (CS) is hypothesized as its pathophysiology, but due to the scarcity of cases, it has yet to be concluded [4, 5]. Though surgical and conservative treatments have been reported, the decision depends on the clinical situation, and the optimal approach has not been determined clearly.

We experienced a rare case of LAD that occurred in total arch replacement (TAR), in which 3D imaging, such as 3D transesophageal echocardiography (TEE) and 3D computed tomography (CT), was valuable to monitor the LAD and determine the treatment strategy.

### Case presentation

A 79-year-old man with severe chronic obstructive pulmonary disease (COPD) was referred to our department for surgical treatment of arteriosclerosis obliterans (ASO) and a 35-mm small abdominal aortic aneurysm (AAA). Enhanced CT revealed the 55-mm true saccular aneurysm in the aortic arch. Informed consent for the treatments and the publication from the patient was obtained.

After 1 month of the aortofemoral bypass for the treatment of AAA and ASO, the patient had total arch replacement (TAR). Following median sternotomy, cardiopulmonary bypass (CPB) was established via superior and inferior vena cava drainage, and right axillary arterial and ascending aortic return. A venting tube was set in the right upper pulmonary vein (RUPV). Cardioplegic cannulae were placed on the aortic root and in the coronary sinus. After aortic cross-clamping, cardiac arrest was inducted by antegrade and retrograde cardioplegia. The initial pressure of the retrograde cardioplegia was as high as 65 mmHg, so its flow was reduced immediately, and the pressure decreased soon. Body temperature was cooled down to 27 °C. After the proximal ascending aorta was anastomosed with a 4-branched artificial graft (J Graft, Japan Lifeline Co., Ltd., Tokyo, Japan), the aortic clamp was released, and deep hypothermic circulatory arrest was commenced.

**Fig. 1:**
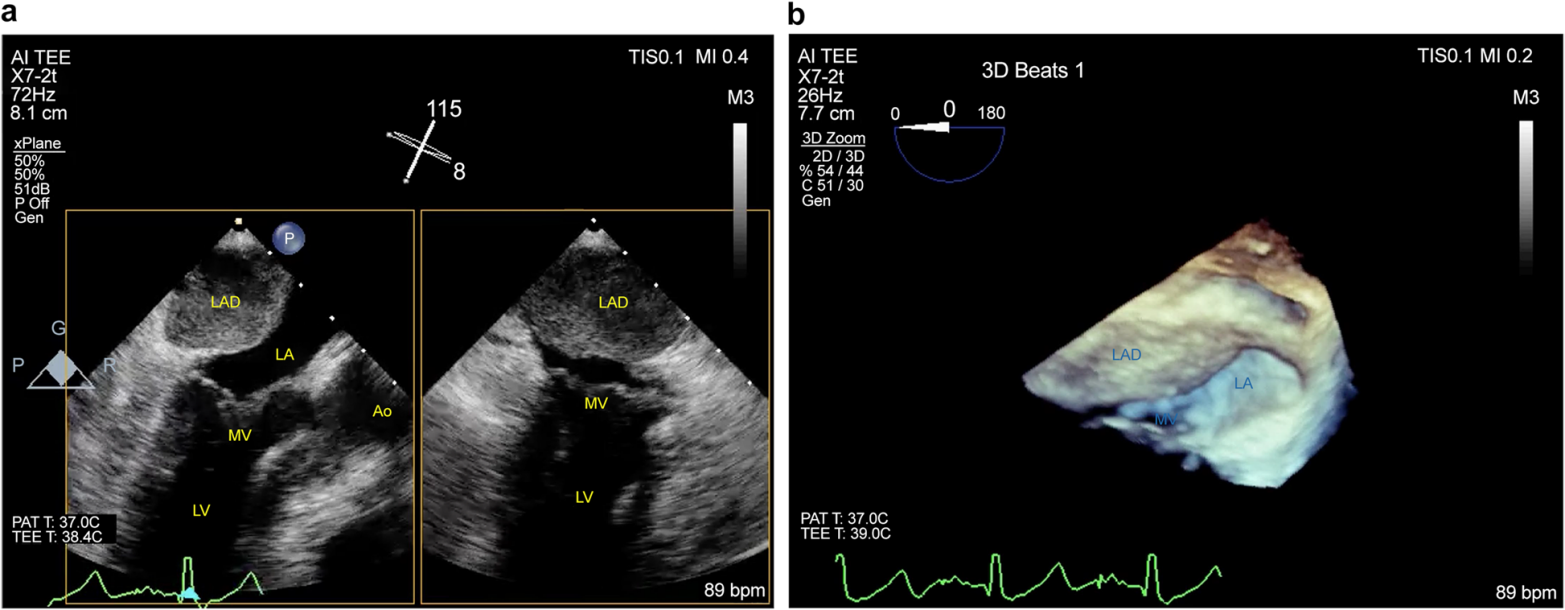
Transesophageal echocardiography (TEE) during total arch replacement (TAR). **a** 2D-TEE exhibited the left atrial dissection (LAD) located at the posterior wall of the left atrium (LA). **b** The LAD and mitral valve (MV) were partly visible on 3D TEE. The LAD contains a thrombus. *Ao* aorta, *LV* left ventricle

A frozen elephant trunk (J Graft FROZENIX, Japan Lifeline Co., Ltd., Tokyo, Japan) was implanted at the distal aortic arch, then distal anastomosis of the 4-branched graft was performed. Then, central perfusion was re-established, body temperature was re-warmed, and cervical branches were re-constructed. While weaning off the CPB, TEE detected LAD (18 × 26 mm) on the posterior wall of the left atrium, and its false lumen was fully contained with hematoma (Fig. 1). Because there was no hemodynamic deterioration, we chose a conservative approach and finished the operation routinely. The patient was extubated on postoperative day (POD) 1 and transferred from ICU to the ward on POD 3.

**Fig. 2:**
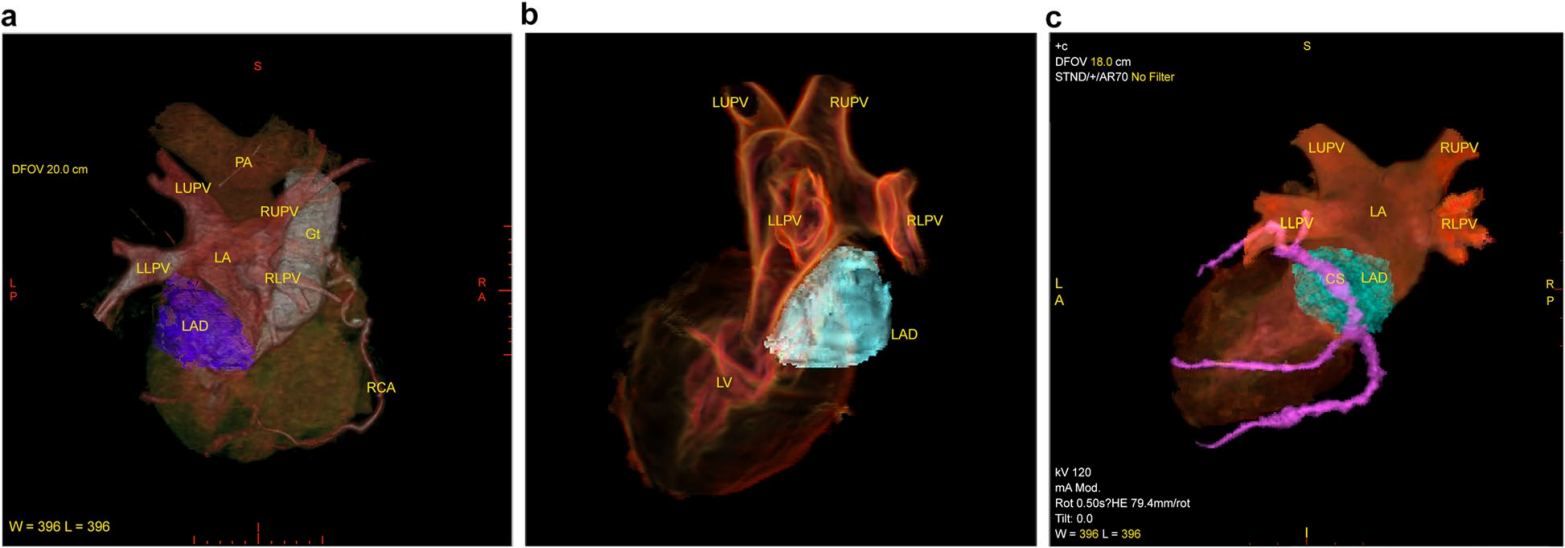
3D computed tomography (CT) identified the shape of the left atrial dissection (LAD) as a blue mass. **a**, **b** There is substantial space between the LAD and the pulmonary veins and **c** the LAD neighbors the coronary sinus. CS coronary sinus, Gt graft, LA left atrium, LAD left atrial dissection, LLPV left lower pulmonary vein, LUPV left upper pulmonary vein, LV left ventricle, PA pulmonary artery, RLPV right lower pulmonary vein, RUPV right upper pulmonary vein, RCA right coronary artery

**Fig. 3:**
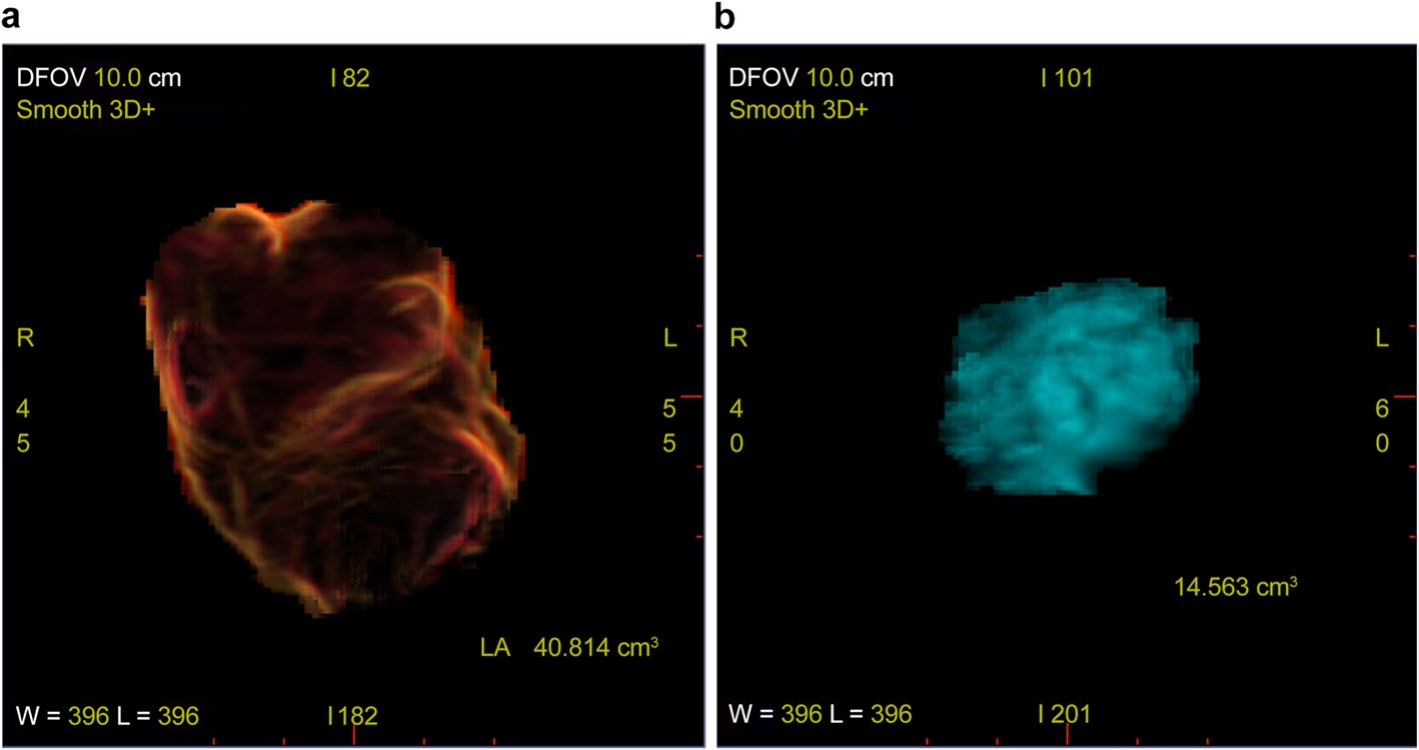
Volume of the left atrium (LA), excluding the left atrial dissection (LAD) (**a**) and the volume of the LAD, which was 40.8 cm^3^ and 14.6 cm^3^, respectively. The LAD occupied 26% of the LA

The extent of the LAD was carefully observed during the postoperative period with sequential transthoracic echocardiography (TTE) on POD 1, 2, 7, 11, and 14. On POD 7, ECG-gated 3D-CT was practiced to exclude obstruction of the pulmonary veins by the LAD. It was performed on a GE Revolution 256-slice scanner. As the LAD mass was enough apart from the pulmonary veins, the whole configuration of the LAD was visible (Fig. 2a, b). The LAD neighbored the CS (Fig. 2c). The volume of the left atrium and the LAD was measured with the CT, which are 55.4 cm^3^ and 14.6 cm^3^, respectively, thereby LAD occupied 26% of the left atrium (Fig. 3).

**Fig. 4:**
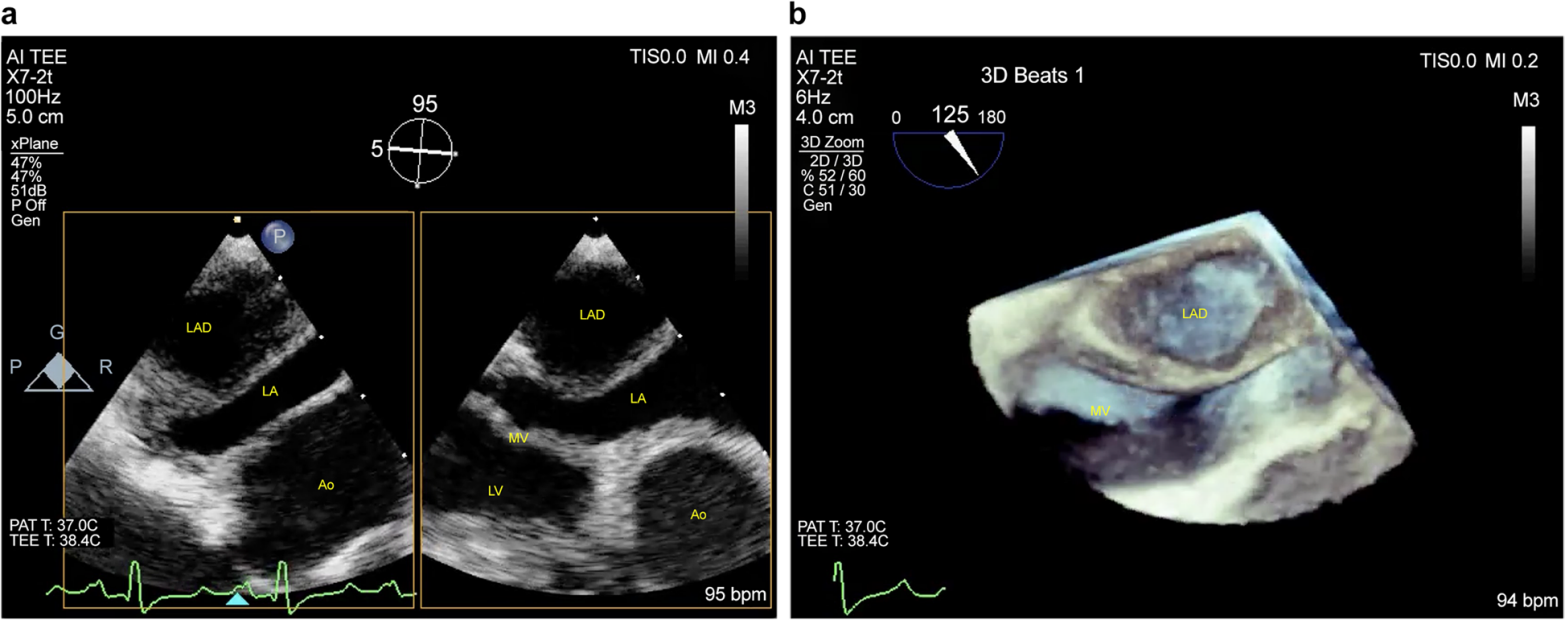
Transesophageal echocardiography (TEE) on postoperative day 15. **a** With the 2D image, the space in the left atrial dissection (LAD) was lower echoic compared with Fig. 1. **b** The area in the LAD was void on 3D TEE because of the hematoma absorption. LA left atrium, LV left ventricle, Ao aorta

Though the volume of the LAD was decreasing on TTEs, delayed tamponade caused the lowering of blood pressure. Then, the pericardial window was performed on POD 15. During this operation, we had an opportunity to perform the second TEE. Hematoma in the false lumen was absorbed, and its space was void with 3D-TEE. The size of LAD was decreased to 14 × 21 mm (Fig. 4). After the pericardial window, blood pressure increased, and he was discharged home at POD 23. During the outpatient follow-up, the LAD disappeared almost entirely after 3 months on TTE.

## Discussion

In this case, we experienced rare LAD, which occurred in the surgical treatment of a thoracic true aortic aneurysm. Therefore, we applied 3D-TEE and 3D-CT for careful follow-up along the challenging postoperative course.

LAD is a rare complication with cardiac surgeries and is mainly reported in mitral valve surgeries [1, 3]. The endocardium is detached from the mitral or tricuspid annular area and spreads to the atrial wall or interatrial septum. Its entity is hypothesized as an injury of the atrioventricular (AV) groove [3] or coronary sinus rupture [5]. It usually locates at the posterior side of the left atrium and may cause hemodynamic disturbance by obstruction of mitral valve inflow or pulmonary vein orifice [3]. We experienced a case of LAD, which occurred during the open repair of the true aortic arch aneurysm. LAD in aortic surgery other than aortic dissection is very rare [1, 4, 6] because in its surgical procedure, either opening the left atrium or suturing the AV groove is not required. In our case, the initial pressure of retrograde cardioplegia was as high as 65 mmHg, so the coronary sinus injury was considered the potential cause. Tolpin reported LAD caused by cannulation to the RUPV [7]. But in our case, unlike their report, the dissected area was away from the pulmonary veins as the 3D-CT exhibited (Fig. 2).

We applied 3D-TEE and 3D-CT in the postoperative period to evaluate the LAD. In addition to hemodynamic parameters, imaging of LAD is also vital to estimate the possibility of hemodynamic deterioration. TEE has been considered the gold standard for assessing the size of LAD [5]. With sequential 3D-TEE, we identified the rapid hematoma absorption inside the LAD. Additionally, to the best of our knowledge, this is the first report that highlights the entire configurative relations of LAD, CS, and pulmonary veins with 3D CT. The proximity between the LAD and CS rationalized the pathophysiology of the LAD in this case. We have also discussed the volume of the LAD because the substantial occupation of the left atrium by thrombosed LAD limits inflow to the left ventricle, which leads to a decrease in preload and hemodynamic collapse. The volume of LAD seemed massive on the TEE; however, 3D CT revealed that its occupancy was only 26% in the left atrium, which was assumed to be acceptable. Furthermore, there was enough space between the LAD and ostia of the pulmonary veins. These findings led us to validate conservative treatment.

The clinical course of LAD is various, and the optimal treatment strategy has yet to be concluded [3, 8, 9]. Both successful surgical and conservative treatments were reported, and the choice depends on each clinical scenario and the natural course of LAD [10–12]. In the present case, two possible causes could have led to the deterioration of the patient’s hemodynamics: LAD and delayed tamponade. However, due to the patient’s fragility, it was crucial to minimize invasive procedures. In such uncertain and complex cases, careful imaging assessment is important in determining the treatment strategy.

As a limitation, the LAD was not exposed in this case not to elongate CPB, so we could not directly identify the fistula between the true and false lumen. However, Martinelli reported communication between the CS and the false lumen of LAD in their autopsy case [5]. In our scenario, it was considered the sole potential cause of the LAD from the clinical findings. Additionally, the threshold of the acceptable volume of the LAD has not been established yet due to the scarcity of the cases and because the acceptable LAD volume depends on each patient’s condition. However, with the accumulation of the data, an approximate guide could be created for collecting more data, which might benefit the other mass diseases occupying the left atrium.

We had a rare case of LAD, which occurred in TAR. Multi-modality 3D imagings, such as 3D-TEE and 3D-CT, were valuable in evaluating the LAD. They helped estimate the pathophysiology and quality of the LAD, which is especially important in complicated, sensitive cases.

## Data Availability

The data that support the findings of this study are available from the corresponding author, upon reasonable request.
